# Inhibition of checkpoint kinase 2 (CHK2) enhances sensitivity of pancreatic adenocarcinoma cells to gemcitabine

**DOI:** 10.1111/jcmm.12101

**Published:** 2013-07-16

**Authors:** Hong-Quan Duong, Young Bin Hong, Jung Soon Kim, Hee-Seok Lee, Yong Weon Yi, Yeon Jeong Kim, Antai Wang, Wenjing Zhao, Chi Heum Cho, Yeon-Sun Seong, Insoo Bae

**Affiliations:** a Department of Oncology, Lombardi Comprehensive Cancer Center, Georgetown UniversityWashington, DC, USA; b WCU (World Class University) Research Center of Nanobiomedical Science, Dankook UniversityCheonan, Korea; c Department of Biostatistics, Herbert Irving Comprehensive Cancer Center, Columbia UniversityNew York, NY, USA; d Department of Obstetrics and Gynecology, Keimyung University School of MedicineDaegu, Korea; e Department of Radiation Medicine, Lombardi Comprehensive Cancer Center, Georgetown UniversityWashington, DC, USA

**Keywords:** Checkpoint kinase 2 (CHK2) inhibitor, NSC109555, gemcitabine, combination, synergism, pancreatic adenocarcinoma

## Abstract

Checkpoint kinase 2 (CHK2) plays pivotal function as an effector of cell cycle checkpoint arrest following DNA damage. Recently, we found that co-treatment of NSC109555 (a potent and selective CHK2 inhibitor) potentiated the cytotoxic effect of gemcitabine (GEM) in pancreatic cancer MIA PaCa-2 cells. Here, we further examined whether NSC109555 could enhance the antitumour effect of GEM in pancreatic adenocarcinoma cell lines. In this study, the combination treatment of NSC109555 plus GEM demonstrated strong synergistic antitumour effect in four pancreatic cancer cells (MIA PaCa-2, CFPAC-1, Panc-1 and BxPC-3). In addition, the GEM/NSC109555 combination significantly increased the level of intracellular reactive oxygen species (ROS), accompanied by induction of apoptotic cell death. Inhibition of ROS generation by N-acetyl cysteine (NAC) significantly reversed the effect of GEM/NSC109555 in apoptosis and cytotoxicity. Furthermore, genetic knockdown of CHK2 by siRNA enhanced GEM-induced apoptotic cell death. These findings suggest that inhibition of CHK2 would be a beneficial therapeutic approach for pancreatic cancer therapy in clinical treatment.

## Introduction

Pancreatic adenocarcinoma is the fourth leading cause of cancer-related deaths in the Western world [1]. In the United States, approximately 43,000 new cases and 37,000 deaths of pancreatic cancer were estimated in 2010 [1]. The overall prognosis of pancreatic cancer patients is extremely poor: the 5-year survival rate is estimated less than 5–6% [2]. Current therapy for pancreatic cancer involved surgery and chemotherapy; however, pancreatic cancer is a notorious disease with resistant to many key chemotherapeutic agents and novel targeted therapies [3]. The front-line standard drug, gemcitabine (GEM), is used for the treatment of advanced human pancreatic cancer either alone or in combination with other chemotherapeutic agents [4–6]. While response rates and progression-free survival have slightly improved, the efficacy is still limited and novel therapies are urgently needed.

Recently, we performed cell viability screenings to identify small molecule kinase inhibitors (PKIs), which potentiate the cytotoxic effect of GEM in parental- and GEM-resistant pancreatic MIA PaCa-2 cell line [7, 8]. Several PKIs, identified from these screening efforts, have been further characterized: (1) blockade of phosphatidylinositol 3-kinase (PI3K)/AKT pathway by ZSTK474 enhances antitumour effect of GEM by inhibiting GEM-induced increase in phospho-AKT and phospho-Bad in human pancreatic cancer Colo-357 and BxPC-3 cell lines [9]; (2) TGFβ receptor I (TβR1) inhibitors, SB431542 and SB525334, sensitize both parental- and GEM-resistant pancreatic cancer cells to GEM by inhibiting activated AKT [10].

Consistent with previous studies [11–14], we also identified checkpoint kinase 1 (CHK1) inhibitors as GEM-sensitizing PKIs in pancreatic cancer MIA PaCa-2 cells [7]. Subsequently, we have further identified a CHK2 inhibitor, NSC109555 [15] as a potentiating agent that could reverse GEM resistance of MIA PaCa-2 cells [8]. CHK1 and CHK2 are structurally and functionally distinct serine/threonine protein kinases both involving in the regulation of cell cycle checkpoints [16]. Although CHK1 activation is primarily dependent on ataxia telangiectasia mutated (ATM) and Rad3-related kinase (ATR) activated by stalled replication forks and single-strand DNA breaks, CHK2 is mainly activated by ATM induced by DNA double-strand breaks (DSBs) [16]. CHK1 controls the intra-S and G2/M checkpoints and CHK2 regulates intra-S, G2/M and G1/S checkpoints respectively [16]. GEM requires phosphorylation to produce its active diphosphorylated (dFdCDP) and triphosphorylated (dFdCTP) metabolites [17, 18]. dFdCDP inhibits ribonucleotide reductase (RNR) to reduce the amounts of deoxynucleotide triphosphate (dNTPs) and dFdCTP competes with dCTP resulting in misincorporation of dFdCTP into DNA [17, 19]. As mentioned, the combination effect of CHK1 inhibitors and GEM has been studied in some detail [11–14]. On the contrary, the effect of CHK2 inhibition in combinatorial treatment has not been investigated. Inhibition of CHK2 expression by antisense CHK2/hCds1 (CHK2AS) reduced DNA damage-induce S and G2 checkpoints and facilitated apoptosis in human embryonic kidney 293 cells (HEK293) in a p53-independent manner [20]. In colorectal cancer HCT116 cell model, accelerated apoptosis was observed in CHK2 knockout cells compared to CHK2 wild-type cells [21]. However, co-treatment of CHK2 inhibitor II antagonized the oxaliplatin-induced cytotoxicity in CHK2 wild-type HCT116 cells [21]. Treatment of C3742, a CHK2 inhibitor, has also been reported to increase efficacy of cisplatin in human ovarian cancer A2780 and Caov-3 irrespective of p53 mutation status [22]. Overexpression of activated CHK2 was observed in both precancerous lesions [23] and several human tumours including bladder, breast and colon tumours [24]. More recently, it has been reported that phosphorylation of CHK2 is elevated in intraductal papillary mucinous neoplasmas of pancreas [25]. Although the level of phospho-CHK2 is gradually decreasing as disease progression, its overall levels are higher in all tumours than in normal controls [25]. In addition, activation of CHK2 in tumour cells by treatment of several chemotherapeutic agents has been reported [26, 27]. These results suggest that CHK2 inhibitor would have a potential therapeutic benefit either as a single agent or an enhancing agent for DNA-targeted agents [16, 26, 27].

In this study, we further investigated the synergistic antitumour effect of NSC109555 (a potent and selective CHK2 inhibitor) in combination with GEM in several pancreatic cancer cells. Our results suggest the potential efficacy of CHK2 to enhance the sensitivity of pancreatic cancer to GEM.

## Materials and methods

### Cell culture and reagents

Pancreatic cancer cell lines (MIA PaCa-2, Panc-1, CFPAC-1 and BxPC-3) and human lung fibroblasts cell line (WI-38) were purchased from American Type Culture Collection (ATCC; Manassas, VA, USA). BxPC-3 cells were cultured in RPMI 1640 medium supplemented with 10% heat-inactivated foetal bovine serum (HI-FBS; HyClone, Logan, UT, USA), 100 units/ml penicillin/streptomycin. MIA PaCa-2 cells were maintained in DMEM containing 10% HI-FBS, 2.5% horse serum (HS), 100 units/ml penicillin/streptomycin. Panc-1 and CFPAC-1 cells were cultured in DMEM containing 10% HI-FBS, 10 units/ml penicillin/streptomycin. WI-38 cells were cultured in ATCC-formulated Eagle's Minimum Essential Medium (EMEM) containing 10% HI-FBS. Table 1 shows the information on the genotype of these cell lines to provide a background for understanding how alterations in these pathways contribute to the growth characteristics, tumorigenicity and chemosensitivity. Cell culture reagents were purchased from BioWhittaker (Walkersville, MD, USA) or Welgene (Daegu, Korea). NSC109555 was purchased from Tocris Bioscience (Bristol, UK) and dissolved in dimethyl sulfoxide (DMSO). GEM was obtained from Sigma-Aldrich (St. Louis, MO, USA) and dissolved in PBS. Small molecule compounds were stored at −20°C in small aliquots.

**Table 1:** Human pancreatic cancer cell lines used in this study

Cell lines	KRAS	TP53	CDKN2A/p16	SMAD4/DPC4
MIA PaCa-2	12 Cys	248 Trp	HD	WT
CFPAC1	12 Val	242 Arg	WT	HD
Panc-1	12 Asp	273 His	HD	WT
BxPC-3	WT	220 Cys	WT	HD

KRAS (v-Ki-ras2 Kirsten rat sarcoma viral oncogene homolog); TP53 (encoding the p53 protein); CDKN2A/p16 (p16 or p16INK4a); SMAD4/DPC4 (SMAD family member 4/deleted in pancreatic carcinoma locus 4).

WT, wild-type; HD, homozygous deletion.

### MTT (3-(4,5-dimethylthiazol-2-yl)-2,5-diphenyltetrazolium bromide) assay

A total of 2000 human pancreatic cancer cells, counted by the Luna Cell Counter (Logos Biosystems, Gyeonggi-Do, Korea), were plated in 96-well flat bottom plates and then treated with GEM and NSC109555 in various concentrations with fixed molar ratio of 1:10 in triplicate. At the indicated times, 20 μl of 1 mg/ml MTT (Sigma-Aldrich) in PBS was added to each well and further incubated for ∼4 hrs. After centrifugation and removal of the medium, 150 μl of DMSO (Sigma-Aldrich) was added to each well to dissolve the formazan crystals. The absorbance was measured at 560 nm using an ELx808 absorbance microplate reader (BioTek Instruments Inc., Winooski, VT, USA). Absorbance of untreated cells was designated as 100% and the relative viable cells were expressed as a percentage of this value. The drug interaction was evaluated using the combination index (CI) according to the method of Chou and Talalay [28] using CompuSyn software (ComboSyn Inc., Paramus, NJ, USA).

### Clonogenic assay

MIA PaCa-2 or Panc-1 cells (2 × 10^5^ cells) were seeded in 60-cm dishes. Twenty-four hours after plating, various concentrations of the drugs, either as single agents or combination, were added to each dish. After treatment for 24 hrs, cells were trypsinized and re-seeded in 60-cm dishes at a density of 500 cells per dish in triplicate. The cells were further incubated for 14 days and stained with 0.5% crystal violet in PBS containing 25% methanol. Colonies were examined under a light microscope and counted after capturing images by scanner. Relative colony numbers were calculated as a percentage of the untreated cells.

### Western blot analysis

Cells were grown to ∼70% confluency and reagents were added as indicated concentrations. Cells were lysed in lysis buffer containing 20 mM Tris-HCl, 0.5 M NaCl, 0.25% Triton X-100, 1 mM EDTA, 1 mM EGTA, 10 mM β-glycophosphate, 10 mM NaF, 300 μM Na_3_VO_4_, 1 mM benzamidine, 2 μM PMSF and 1 mM DTT. The protein concentration was determined by the BCA protein assay (Thermo Scientific, Rockford, IL, USA). Proteins were separated on SDS-PAGE, transferred on to PVDF membrane, blocked in 1× blocking buffer (Sigma-Aldrich) and probed with the following antibodies: phospho-CHK2 (T68), phospho-CHK2 (S516), phospho-ATM (S1981), phospho-ATR (S428), phospho-CHK1 (S296), phospho-Histone H2A.X (S139) and Poly-ADP-Ribose-Polymerase (PARP; Cell Signaling Technology, Boston, MA, USA), phospho-Cdc25A (S79) and CHK2 (Santa Cruz Biotechnology, Santa Cruz, CA, USA), and α-tubulin (Sigma-Aldrich). Then, the membranes were incubated with horseradish peroxidase (HRP)-conjugated secondary antibodies (Sigma-Aldrich), incubated with a chemiluminescence reagent (Santa Cruz Biotechnology) according to the manufacturer^'^s recommendation and exposed with X-ray film (American X-ray & Medical Supply, Jackson, CA, USA) [9].

### Caspase-3/7 activity assay

Caspase-3/7 activity assay was carried out using a Caspase-3/7 Glo Assay (Promega, Madison, WI, USA) according to the manufacturer^'^s protocol. MIA PaCa-2 and BxPC-3 cells were treated with either GEM, NSC109555 alone, or combination of both drugs for indicated times and caspase-3/7 activity was measured from cell lysates. Luminescence was measured at 490 nm using VICTOR X multilable plate readers (Perkin-Elmer Life Sciences, Boston, MA, USA). Relative luminescence units were determined by calculating luminescence values from samples as a percentage of values from vehicle-treated control samples. The experiments were performed in triplicate and repeated on two separately initiated cultures.

### Annexin V/Propidium Iodide assay

For Annexin V/Propidium Iodide (PI) assay, cells were stained with FITC-conjugated Annexin V/PI and evaluated for apoptosis by flow cytometry according to the manufacturer^'^s protocol (BD PharMingen, San Diego, CA, USA). Briefly, MIA PaCa-2 cells were treated with either GEM, NSC109555 alone, or combination of both drugs for 24 hrs. The apoptotic cells were analysed on a FACSCalibur flow cytometer (Becton Dickinson, San Jose, CA, USA) at the Flow Cytometry & Cell Sorting Shared Resource, Georgetown University Lombardi Comprehensive Cancer Center.

### Measurement of ROS production

MIA PaCa-2 cells were treated with either GEM, NSC109555 alone, or combination of both drugs in the presence or absence of N-acetyl cysteine (NAC) for the indicated times and then loaded with 50 μM 2′,7′-dichlorofluorescin diacetate (DCFDA; Molecular Probes, Eugene, OR, USA) and 0.5 μg/ml Hoechst 33342 (HO; Sigma-Aldrich) for 30 min. After thorough rinsing, fluorescence intensities were obtained with a Fluorocount at excitation/emission wavelengths of 490/530 nm (DCFDA) and 340/425 nm (HO) using VICTOR X multilable plate reader, and then values of reactive oxygen species (ROS) were obtained by determining the ratio of DCFDA/HO signals per well [29].

### Small interfering RNA (siRNA)

CHK2-siRNA, 5′-CCUGUGGAGAGGUAAAGCU-3′ and control-siRNA, 5′-GACGAGCGGCACGUGCACA-3′ were purchased from Bioneer (Daejeon, Korea). These siRNA were transfected into MIA PaCa-2 cells using Lipofectamine™ 2000 (Invitrogen, Carlsbad, CA, USA) according to the manufacture^'^s procedure. After 48 hrs, transfected cells were processed for WB analysis, caspase-3 activity, Annexin V/PI assay, ROS measurement and MTT cell proliferation assay.

### Statistical analysis

The two-tailed Student's *t*-test was applied for statistical analysis when only two groups of interest were compared. For comparisons with multiple groups, one-way or two-way anova were implemented. After the overall analysis was performed for each data set, Turkey tests controlling the type one error have been performed to make the pairwise comparisons between the treatment groups. Results were considered significant in all experiments at * means *P* < 0.05, ** means *P* < 0.01 and *** means *P* < 0.001.

## Results

### NSC109555 potentiates the cytotoxicity of GEM in pancreatic cancer cells

**Fig. 1:**
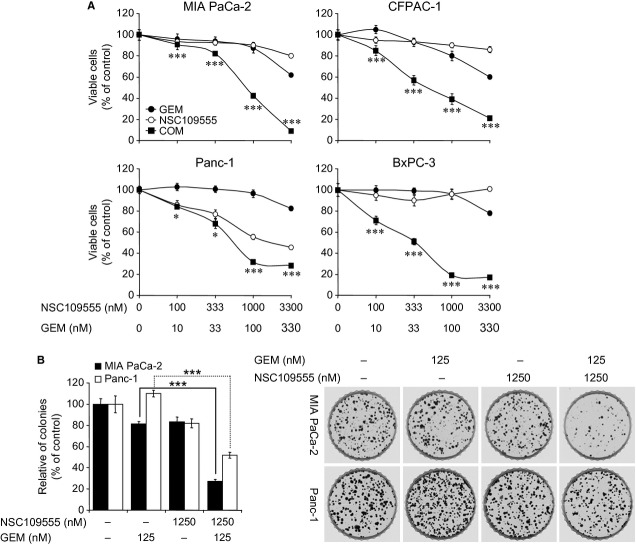
The synergistic antitumour effect by combination treatment of NSC109555 and gemcitabine (GEM). (**A**) Pancreatic cancer cells were co-treated with NSC109555 and GEM with fixed molar ratio of 10:1 for 72 hrs and viable cells were determined by MTT assay. Data from two independent experiments performed in triplicate are shown as mean ± SD. (**B**) MIA PaCa-2 and Panc-1 cells were treated as indicated for 24 hrs and then the long-term responses were determined by clonogenic assay. Experiments were repeated three times and similar results were obtained. Data are shown as mean ± SD. *P < 0.05; **P < 0.01; ***P < 0.001.

Previously, we identified that a small molecule CHK2 inhibitor, NSC109555 [15], sensitizes GEM-resistant MIA PaCa-2 cells to GEM [8]. It has been also reported that inhibition of CHK2 activity by selective CHK2 inhibitors enhanced the cytotoxic effects of several chemotherapeutic agents [30], Topoisomerase I inhibitors [31] and PARP inhibitors [32]. Given these results, we examined whether inhibition of CHK2 would enhance the sensitivity of pancreatic cancer cells to GEM. Pancreatic cancer cells (MIA PaCa-2, CFPAC-1, Panc-1 and BxPC-3) and human lung fibroblasts cells (WI-38) were simultaneously treated with either each drug alone or combination of both drugs for 72 hrs in fixed molar ratio of 10:1. As shown in Figure 1A, NSC109555 potentiated the cytotoxicity of GEM in all pancreatic cancer cell lines tested. However, WI-38 did not show the cytotoxicity by combination treatment of NSC109555/GEM (Figure S1). To confirm that NSC109555 synergized the effect(s) of GEM, we calculated the CI with a range of concentrations of NSC109555 and GEM using the CalcuSyn software program. Table 2 shows that the GEM/NSC109555 combination showed synergistic anti-proliferative effects to all cell lines tested in a broad range with CI values at ED_50_, ED_75_ and ED_90_ with 0.10, 0.06 and 0.04 in MIA PaCa-2 cells, with 0.09, 0.14 and 0.22 in CFPAC-1 cells, with 0.42, 0.38 and 0.47 in Panc-1 cells and with 0.06, 0.08 and 0.11 in BxPC-3 cells respectively. Combination of NSC109555 and GEM exhibited the strong synergism in Mia PaCa-2, CFPAC-1 and BxPC-3 cells, and less synergism in Panc-1 cells (Fig. 1A). The effect of GEM/NSC109555 combination was further evaluated in long-term clonogenic assays. MIA PaCa-2 and Panc-1 cells treated with either single agents or GEM/NSC109555 combination for 24 hrs and the cells were re-seeded and continually cultured in normal growth media for 14 days. Under this condition, GEM or NSC109555 minimally affected the colony-forming ability of these cells (Fig. 1B). However, cell survivals were profoundly reduced in both MIA PaCa-2 and in Panc-1 when cells were simultaneously treated with NSC109555 and GEM (Fig. 1B). Taken together, these results suggest that the combinatorial treatment of NSC109555 and GEM results in a synergistic inhibition of the cell proliferation and the colony-forming potential of pancreatic cancer cells.

**Table 2:** Synergism of gemcitabine/NSC109555 combination in human pancreatic cancer cells. Table 1 shows CI values obtained from experiments using the MIA PaCa-2, CFPAC-1, Panc-1 and BxPC-3 cells. These CI values were calculated by the Chou and Talalay method for drug interactions using Compusyn software for the different fractions affected (the CI values at ED_50_, ED_75_ and ED_90_). Values of CI<1, =1 and >1 indicate synergism, additive effects and antagonism respectively

	Combination index (CI)		
	
	ED_50_	ED_75_	ED_90_
MIA PaCa-2	0.10	0.06	0.04
CFPAC1	0.09	0.14	0.22
Panc-1	0.42	0.38	0.47
BxPC-3	0.06	0.08	0.11

### NSC109555 enhances apoptotic cell death induced by GEM

**Fig. 2:**
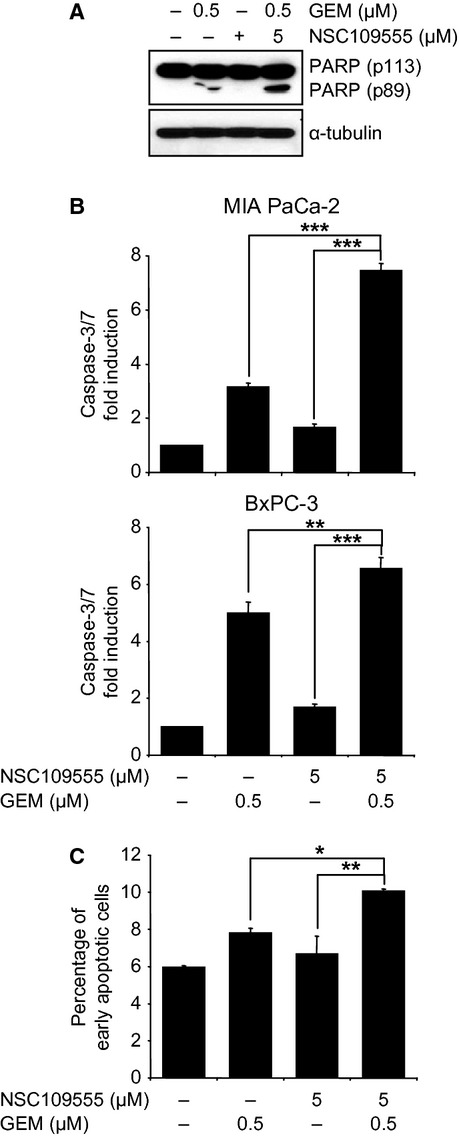
NSC109555 enhances gemcitabine (GEM)-induced apoptotic cell death. (**A**) MIA PaCa-2 cells were treated either with 5 μM NSC109555, 0.5 μM GEM alone or combination of both drugs for 48 hrs. The western blot analysis was performed to detect poly-ADP-ribose-polymerase cleavage as a marker for apoptotic cell death. The α-tubulin was used as the loading and transfer control. (**B**) MIA PaCa-2 and BxPC-3 cells treated as described in (**A**) were lysed and caspase-3/7 activities were measured as described in Materials and methods. (**C**) MIA PaCa-2 cells were treated with 5 μM NSC109555, 0.5 μM GEM alone or combination of both drugs for 24 hrs. Apoptotic cells were detected by annexin V/PI staining as described in Materials and methods. Representative data are shown as mean ± SD from three independent experiments. *P < 0.05; **P < 0.01; ***P < 0.001.

To further assess the synergism by NSC109555 in pancreatic cancer cells, we performed western blot analysis to detect the change in an apoptotic marker, PARP. MIA PaCa-2 cells co-treated with 5 μM NSC109555 and 0.5 μM GEM for 48 hrs and subjected to western blot analysis. Under this condition, NSC109555 did not induce PARP cleavage, whereas GEM induced significant amount of PARP cleavage. Comparing with 0.5 μM GEM treatment, NSC109555/GEM treatment further increased the GEM-induced cleavage of PARP (Fig. 2A). Consistent with PARP cleavage, GEM alone induced significant amount of caspase-3/7 activity than NSC109555 did as a single agent. In addition, co-treatment of NSC109555 markedly enhanced the GEM-mediated caspase-3/7 activity in MIA PaCa-2 and BxPC-3 cells (Fig. 2B). Accordingly, analysis of annexin V/PI staining revealed an increase in early apoptotic cells (Annexin V^+^/PI^−^) only after NSC109555/GEM treatment in MIA PaCa-2 cells (Fig. 2C). These results suggest that a specific blockade of CHK2 activity by NSC109555 significantly enhances GEM-induced apoptotic cell death *via* activation of caspase-3/7 and PARP cleavage in pancreatic cancer cells.

### NSC109555 decreases GEM-induced CHK2 phosphorylation

**Fig. 3:**
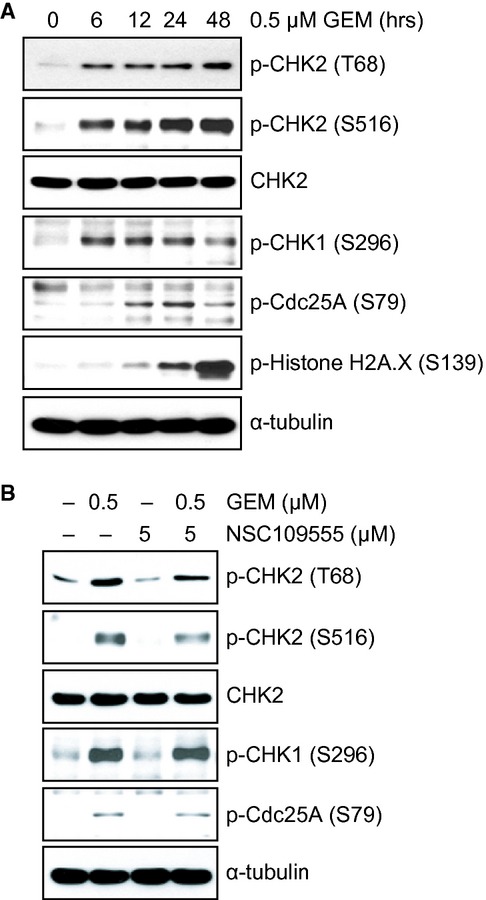
NSC109555 decreases gemcitabine (GEM)-induced CHK2 phosphorylation. Western blot analysis was performed with indicated antibodies in cell lysates from (**A**) MIA PaCa-2 cells treated with 0.5 μM GEM for indicated times and (**B**) MIA PaCa-2 cells treated as indicated for 24 hrs. α-tubulin was used for a loading and transfer control.

Phosphorylation of CHK2 has been known to be increased by treatment with several chemotherapeutic agents [26, 27]. Accordingly, we observed that treatment of 0.5 μM GEM substantially increased the CHK2 phosphorylation (T68) and autophosphorylation (S516) without change in total CHK2 protein level in MIA PaCa-2 cells (Fig. 3A). GEM-induced CHK2 phosphorylation (T68) and autophosphorylation (S516) was evident as early as at 6 hrs after treatment and sustained to 48 hrs after treatment (Fig. 3A). In addition, treatment of GEM also increased the level of phospho-Cdc25A (S79), a well known substrate of CHK2, in a time-dependent manner and maximal induction was observed at 24 hrs after treatment (Fig. 3A). To determine the effect of NSC109555 in GEM-induced CHK2 phosphorylation and autophosphorylation, cells were treated with either 5 μM NSC109555 or 0.5 μM GEM alone, or combination of both drugs for 24 hrs. As expected, NSC109555 significantly suppressed GEM-induced CHK2 phosphorylation (T68) and autophosphorylation (S516) (Fig. 3B). Furthermore, NSC109555 partly suppressed GEM-induced phosphorylation level of Cdc25A (S79) (Fig. 3B). To confirm the specificity of NSC109555, we checked the phosphorylation status of CHK1 (S296) (Fig. 3A and B), ATM (S1981) and ATR (S428) (Figure S2A and B). As shown in Figure 3A and Figure S2A, accumulation of phospho-CHK1 (S296) and phospho-ATR (S428) was markedly increased by treatment of GEM at 6 hrs and slightly decreased in time-dependent manner; and accumulation of phospho-ATM (S1981) was increased at 6 hrs and sustained to 48 hrs after treatment. We also checked the phosphorylation level of Histone H2A.X (S139), a well-known marker of DNA damage. Consistent with accumulation of the CHK2 phosphorylation (T68) and autophosphorylation of CHK2 (S516), the level of phospho-Histone H2A.X (S139) was dramatically increased in time-dependent manner (Fig. 3A). However, NSC109555 did not suppress the GEM-induced phosphorylation of CHK1 (S296) (Fig. 3B), ATM (S1981) and ATR (S428) (Figure S2B). These results implicated that NSC109555 specifically inhibited the phosphorylation and autophosphorylation of CHK2 under these conditions.

### Apoptotic cell death induced by GEM/NSC109555 combination is dependent on ROS

**Fig. 4:**
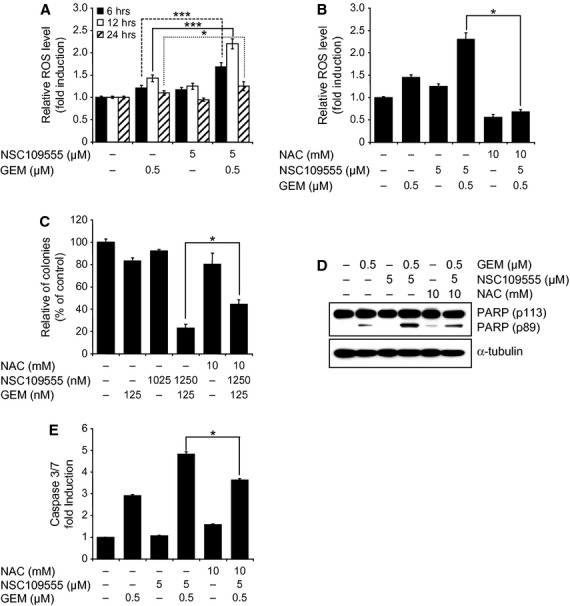
Inhibition of reactive oxygen species (ROS) production reduces gemcitabine (GEM)/NSC109555 combination–mediated apoptotic cell death. (**A**) The measurement of ROS generation in MIA PaCa-2 cells treated as indicated in different time intervals. The level of ROS was measured as described in Materials and methods. (**B**) MIA PaCa-2 cells were pre-treated with 10 mM NAC for 1 hr and further treated as indicated for 12 hrs and the level of ROS was measured as (**A**). (**C**) MIA PaCa-2 cells were pre-treated with 10 mM NAC for 1 hr and further treated as indicated for 24 hrs and the long-term effects were determined by colony formation. (**D**) MIA PaCa-2 cells were pre-treated with 10 mM NAC for 1 hr and further treated as indicated for 48 hrs and western blot analysis was performed with poly-ADP-ribose-polymerase antibody. α-tubulin was used for a loading and transfer control. (**E**) MIA PaCa-2 cells, treated as described in (**D**), were lysed and caspase-3/7 activities were measured as described in Materials and methods. (**A**–**C** and **E**) Representative data are shown as mean ± SD from three independent experiments. *P < 0.05; **P < 0.01; ***P < 0.001.

It is well known that GEM induces ROS generation and the sensitivity of pancreatic cancer cells to GEM is related to intracellular ROS stress [33]. We next investigated whether NSC109555 could enhance GEM-induced ROS production in pancreatic cancer cells. Low concentration of GEM (0.5 μM) alone slightly induced ROS at 12 hrs after treatment, while NSC109555 (5 μM) showed little or no effect on ROS generation. On the contrary, co-treatment of GEM with NSC109555 markedly enhanced the ROS generation as early as 6 hrs after treatment (Fig. 4A). Enhancement of GEM-induced ROS generation by NSC109555 was significantly diminished by pre-treatment with the antioxidant agent, NAC (Fig. 4B). NAC also rescued the inhibition of cell survival by GEM/NSC109555 combination in both short-term MTT (data not shown) and long-term clonogenic assay (Fig. 4C). NSC109555/GEM-induced cleavage of PARP and caspase-3/7 activity was also relieved by NAC pre-treatment (Fig. 4D and E). To determine the direct correlation between ROS and apoptosis or other phenomena could mediate the effects of ROS on cell survival, we performed FACS analysis and examined changes in cell cycle progression when MIA PaCa-2 and BxPC-3 cells were co-treated with 5 μM NSC109555 and/or 0.5 μM GEM for 48 hrs. The results showed that GEM markedly induced S-phase and reduced G2/M-phase accumulation; NSC109555 slightly induced G0/G1-phase and reduced S-phase. However, cells treated with combination of NSC109555 and/or GEM did not show significant difference of cell cycle distribution (data not shown). As far as we know that cells treated with combination of NSC109555 and/or GEM markedly induced ROS generation and apoptotic cell death, the result implicated that there may be a direct correlation between ROS generation and apoptosis. Taken together, these results suggest that anti-proliferative effect of NSC109555/GEM combination is, at least, partially dependent on the ROS generation in pancreatic cancer cells.

### CHK2 knockdown enhances apoptotic cell death induced by GEM

**Fig. 5:**
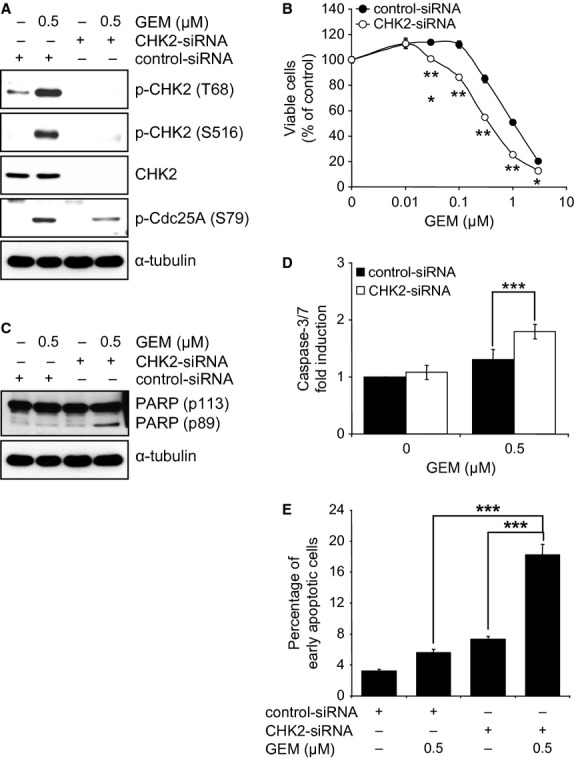
Knockdown of CHK2 enhances gemcitabine (GEM)-induced apoptotic cell death. (**A**) MIA PaCa-2 cells were transfected with either CHK2-siRNA or control-siRNA for 48 hrs and further treated with 0.5 μM GEM for 48 hrs. Western blot analysis was performed with indicated antibodies. α-tubulin was used for a loading and transfer control. (**B**) MIA PaCa-2 cells were transfected with either CHK2-siRNA or control-siRNA for 48 hrs and further treated with GEM for 72 hrs and the viable cells were measured by MTT assay. Data are shown as mean ± SD. **P < 0.01. (**C**) The cell lysates from (**A**) were used to detect PARP and PARP-cleaved forms by Western blot assay. (**D**) MIA PaCa-2 cells were treated as described in (**C**) and caspase-3/7 activities were measured. (**E**) MIA PaCa-2 cells were treated as described in (**C**) and apoptotic cells were detected by annexin V/PI staining. Representative data are shown as mean ± SD from three independent experiments. ***P < 0.005.

To exclude potential off-target effect of NSC109555, we further tested the effect of CHK2 knockdown on GEM-induced cell death in MIA PaCa-2 cells. Knockdown of CHK2 by CHK2-specific siRNA completely reduced the level of CHK2 in MIA PaCa-2 cells (Fig. 5A). Under this condition, GEM-induced accumulation of the CHK2 phosphorylation (T68) and autophosphorylation (S516) was also abolished by CHK2-siRNA. Consistent with NSC109555 treatment (Fig. 3B), knockdown of CHK2 suppressed the phosphorylation level of Cdc25A (S79). The cytotoxic effect of GEM was also assessed in MIA PaCa-2 cells with CHK2 knockdown. MIA PaCa-2 cells, pre-treated with CHK2-siRNA or control-siRNA for 48 hrs, were further incubated with a various concentrations of GEM for 72 hrs and cell viability was determined by MTT assay. As shown in Figure 5B, knockdown of CHK2 by siRNA sensitized the cells to GEM. In addition, GEM-induced PARP cleavage and caspase-3/7 activity were enhanced in MIA PaCa-2 cells treated with CHK2-siRNA than in Mia PaCa-2 cells treated with control-siRNA (Fig. 5C and D). Furthermore, analysis of annexin V/PI staining revealed an increase in early apoptotic cells (Annexin V^+^/PI^−^) in MIA PaCa-2 cells treated with CHK2-siRNA plus GEM than in MIA PaCa-2 cells treated with control-siRNA plus GEM (Fig. 5E).

## Discussion

In this study, we investigated the antitumour effect of combinatorial treatment of NSC109555 (a selective CHK2 inhibitor) and GEM in pancreatic adenocarcinoma cells. We found that (*i*) a synergistic antitumour effect is achieved by GEM/NSC109555 combination in MIA PaCa-2, CFPAC-1, Panc-1 and BxPC-3 cells; (*ii*) NSC109555 inhibits GEM-induced CHK2 phosphorylation; (*iii*) the GEM/NSC109555 combination–induced apoptotic cell death is at least partially dependent on ROS generation; and (*iv*) knockdown of CHK2 potentiates apoptotic cell death by GEM. These results strongly support the hypothesis that inhibition of CHK2 phosphorylation could enhance the sensitivity of pancreatic cancer cells to GEM. To our best knowledge, this is the first report showing the synergistic antitumour effect by combinatorial treatment of GEM with a CHK2 inhibitor in pancreatic cancer cells.

Although CHK2 is a potential target for cancer therapy, studying the effect of CHK2 inhibition is hampered by limited number of specific CHK2 inhibitors [16]. Phosphorylation of CHK2 is induced by treatment of anticancer agents including doxorubicin [34], etoposide [34, 35], GEM [36], cisplatin [37] and ionizing radiation [38]. The underlying mechanism of CHK2 activation by these agents is still controversial and remains to be elucidated [34–38]. As reported in other cell models [36], GEM induced the CHK2 phosphorylation (T68) and autophosphorylation (S516) in MIA PaCa-2 cells. The T68 residue is known to be the priming phosphorylation site that is required to cause homodimerization and subsequent *trans*-activating autophosphorylations (T383 and T387) and *cis*-activating autophosphorylation (S516) to activate down-stream signalling events [39, 40, 27]. As shown in this report, NSC109555 significantly antagonizes the GEM-induced CHK2 phosphorylation (T68) and autophosphorylation (S516), partly suppressed GEM-induced phosphorylation of Cdc25A (S79), the true known substrate of CHK2 [27] and enhances the GEM-induced antitumour effects in pancreatic cancer cells.

Gemcitabine remains the most commonly used chemotherapeutic agent to treat pancreatic cancer [4–6]. However, chemoresistance of patients, both innate and acquired, to GEM has been a major concern with very low response rate [4]. Many attempts are on the way to improve the efficacy of GEM by various combinations of other drugs or novel targeted therapeutics [41]. GEM induced the cellular ROS and subsequently caused apoptotic cell death in pancreatic cancer cells [41, 42]. However, precise mechanism for GEM-mediated ROS generation remains to be determined. As demonstrated in this study, submicromolar concentration of GEM induced ROS production in MIA PaCa-2 cells. Although NSC109555 itself did not induce ROS level, co-treatment of NSC109555 profoundly enhanced the GEM-induced ROS production in MIA PaCa-2 cells. The importance of ROS production resulting from GEM/NSC109555 combination was further emphasized by demonstrating that suppression of ROS generation by antioxidant NAC reduced the long-term cytotoxic effect and apoptotic cell death caused by this combination. Recent advances in cancer biology have shed light on the role of ROS in cancer chemotherapy [43]. It remains largely unknown how CHK2 inhibition increases ROS production induced by DNA damaging agents such as GEM. ATM knockout mice study suggests that checkpoint components can have some function to detoxify ROS induced by DNA damage [44].

It is not easy to demonstrate whether CHK2 inhibition should be more or less effective than CHK1 inhibition as a therapeutic approach for pancreatic cancer. Previous studies demonstrated that inhibition of CHK1 by CHK1 inhibitor sensitizes pancreatic cancer to GEM by mechanisms including G2 checkpoint abrogation and homologous recombination repair inhibition [12, 45, 46]; In addition, inhibition of CHK1 by siRNA also enhances the sensitivity of GEM in pancreatic cancer cells [11]. On the contrary, no previous study showed the therapeutic effects of CHK2 inhibition when combination with GEM. As far as we know, our study is the first report demonstrating that inhibition of CHK2 by either CHK2 inhibitor or siRNA sensitizes pancreatic cancer cells to GEM. Based on previous studies and our new studies, we believe that both CHK1 and CHK2 may be promising therapeutic targets to overcome the resistance of GEM in pancreatic cancer. Further studies will be needed to demonstrate the molecular mechanism of CHK1 and CHK2 inhibition as new potential therapeutic target approaches when combination with GEM in pancreatic cancer.

## Conclusion

Our study shows that NSC109555, a selective CHK2 inhibitor, significantly enhances the antitumour effect when combined with GEM in pancreatic cancer cells. Our finding may provide a novel strategy for the preclinical and clinical application of this combination in pancreatic cancer.

## Supporting information

**Figure S1.** NSC109555 do not enhance the sensitivity of human lung fibroblasts WI-38 cells to gemcitabine (GEM).

**Figure S2.** NSC109555 do not decrease gemcitabine (GEM)-induced ATM and ATR phosphorylation.
