# Supplementary material for: Inhibition of checkpoint kinase 2 (CHK2) enhances sensitivity of pancreatic adenocarcinoma cells to gemcitabine
Source: J Cell Mol Med. 2013 Jul 16;17(10):1261–70. doi: 10.1111/jcmm.12101 (PMC4159025; doi:10.1111/jcmm.12101)
Supplement: Supplementary file 1 [file jcmm0017-1261-SD1.doc]

**Supplementary Figure Legends**

**Supplementary Figure 1. NSC109555 do not enhance the sensitivity of human lung fibroblasts WI-38 cells to gemcitabine (GEM).** WI-38 cells were co-treated with NSC109555 and GEM with fixed molar ratio of 10:1 for 72 h and viable cells were determined by MTT assay. Data from two independent experiments performed in triplicate are shown as mean ± SD.

**Supplementary Figure 2. NSC109555 do not decrease gemcitabine (GEM)-induced ATM and ATR phosphorylation.** Western blot analysis was performed with indicated antibodies in cell lysates from (**A**) MIA PaCa-2 cells treated with 0.5 μM GEM for indicated times and (**B**) MIA PaCa-2 cells treated as indicated for 24 h. α-tubulin was used for a loading and transfer control.
