# Supplementary figures and images for: Inhibition of checkpoint kinase 2 (CHK2) enhances sensitivity of pancreatic adenocarcinoma cells to gemcitabine
Source: J Cell Mol Med. 2013 Jul 16;17(10):1261–70. doi: 10.1111/jcmm.12101 (PMC4159025; doi:10.1111/jcmm.12101)

# Supplementary Figure S1

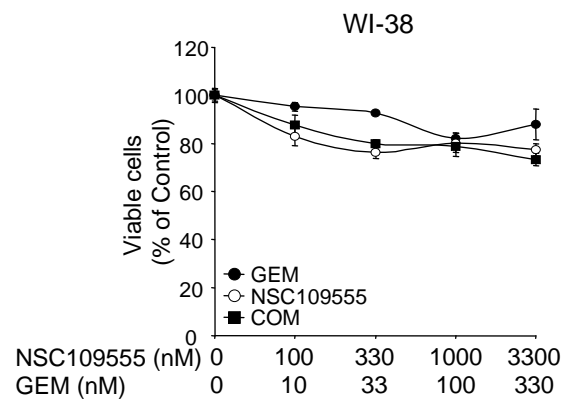

## Supplementary Figure S2

**A**

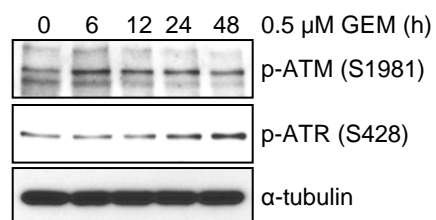

**B**

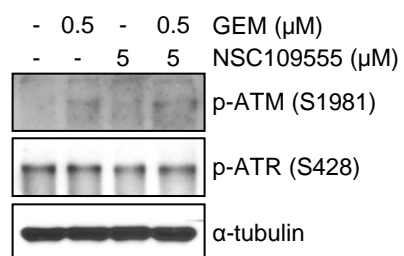

Supplement: Supplementary file 2 [file jcmm0017-1261-SD2.pdf]
